# Using R to improve rigour and transparency in translational neuroscience—or is it just a rabbit hole?

**DOI:** 10.1093/braincomms/fcab290

**Published:** 2022-01-25

**Authors:** Tara L. Spires-Jones

**Affiliations:** Centre for Discovery Brain Sciences, UK Dementia Research Institute, University of Edinburgh, Edinburgh, UK

## Abstract

Our Editor explores the use of R for improving rigour, reproducibility and transparency in translational neuroscience. She also provides an example of how to waste enormous amounts of time playing with data in R instead of doing her day job.

## Graphical Abstract

**Graphical Abstract:**
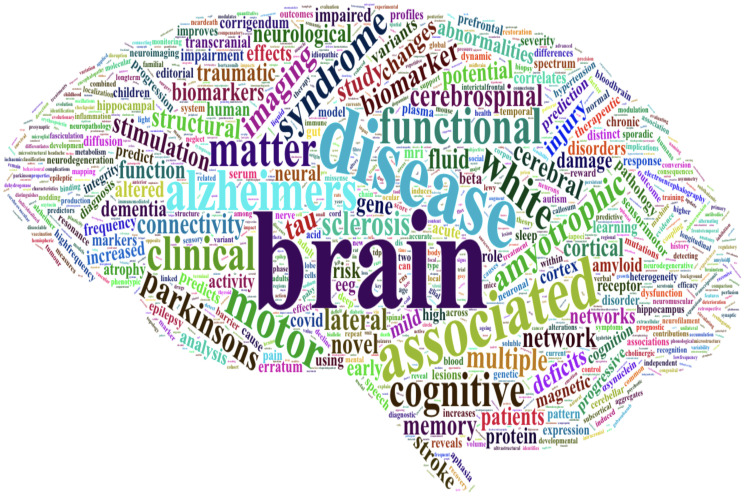

This semester, my life seems consumed by R. For those of you who have not used it, R is a free programming environment for statistical analysis and data visualization.^1^ I came across R in my long, slow effort to improve the statistical analyses of our lab's data. We often take multiple measurements per animal or human tissue donor and wanted to be able to analyse all of our data points without treating these multiple measures as independent (called psuedoreplication).^2^ For years, we had been taking a mean or median from each subject, but this approach loses a lot of the within-subject nuance and variability, so I finally bit the bullet and learned a little about linear mixed-effects modelling, and how to do this in R. This was a painful process to begin with because of my limited knowledge of biostatistics and fear of programming. However, thanks to the patience of fantastic PhD students and biostatistics colleagues, it turned out to be transformative for our lab. We now have R script pipelines for all of our data analysis, including generating graphs and tables. These are very easy to apply to future projects using similar data and allow for easy collaboration between lab members to validate each other's work. We are now sharing our scripts and underlying data files as supplemental information with all the papers we are submitting in an effort to enhance data reuse and transparency. Often when authors at *Brain Communications* fall foul of our policy not to publish pseudoreplicated data, I share these scripts, which are simple to apply once you get the hang of it (available on GitHub alongside all of our other in house generated scripts/software).^3^

In addition to my own work, I have spent a lot of time this semester teaching our honours neuroscience undergraduate students how to use R for analysing data. We are trying to instil in our early career researchers a strong foundation in rigorous data analysis to help enhance credibility in translational neuroscience. There is some great work being done in this area by the British Neuroscience Association if you would like to find out more.^4^

The downsides to using R for me are that learning programming is daunting, generating graphs the way you want them is hugely annoying and complex in R, and most importantly, using R to look at data is strangely addictive to me. On many days and nights, I have found myself spending hours down the R rabbit hole. For example, I have what seems like a million things on my to-do list today, but did I spend the morning learning to import data from *Brain Communications* into R from PubMed in order to make a pretty word cloud for this Editorial instead? Yes, yes I did (see Graphical abstract).

We would love to hear your thoughts on whether R is a useful tool for enhancing credibility in translational neuroscience. Tell us on Twitter @BrainComms or submit a Field Potential article on the topic if you are interested.

The cover image for this issue is a word cloud generated from our article titles pulled from PubMed in R. Many thanks to Dr Phill Jones, who had to fix my code late one night to get it into the shape of a brain. That is R for you!
